# Pegylated versus non-pegylated interferon beta 1a in patients with relapsing-remitting multiple sclerosis: A cost-effectiveness analysis

**Published:** 2018-07-06

**Authors:** Amir Hashemi-Meshkini, Hedieh Sadat Zekri, Hasan Karimi-Yazdi, Pardis Zaboli, Mohammad Ali Sahraian, Shekoufeh Nikfar

**Affiliations:** 1 Department of Pharmacoeconomics and Pharmaceutical Administration, School of Pharmacy, Tehran University of Medical Sciences, Tehran, Iran; 2 Department of Health Economics and Management, School of Public Health, Tehran University of Medical Sciences, Tehran, Iran; 3 School of Medicine, Qom University of Medical Sciences, Qom, Iran; 4 Multiple Sclerosis Research Center, Neuroscience Institute, Tehran University of Medical Sciences, Tehran, Iran

**Keywords:** Interferon Beta-1a, Multiple Sclerosis, Cost-Benefit Analysis, Iran

## Abstract

**Background:** Pegylated (PEG) interferon beta 1a has been approved by the United States Food and Drug Administration (USFDA) as an alternative to interferon beta 1a for multiple sclerosis (MS). Due to its higher price, this study aimed to evaluate the cost-effectiveness of PEG-interferon beta 1-a compared with interferon beta 1a from an Iranian payer perspective.

**Methods:** A Markov model was designed according to health states based on Expanded Disability Status Scale (EDSS) and one-month cycles over a 10-year time horizon. Direct medical and non-medical costs were included from a payer perspective.

**Results:** The incremental cost-effectiveness ratio (ICER) was estimated around 11111 US dollars (USD) per quality-adjusted life-year (QALY) gained for the PEG-interferon versus interferon regimen [with currency rate of 29,000 Iranian Rial (IRR) to 1 USD in 2016].

**Conclusion:** Considering the cost-effectiveness threshold in Iran [three times of gross domestic product (GDP) per capita or 15,945 USD], PEG-interferon beta 1-a could be considered as a cost effective treatment for Iranian patients with MS.

## Introduction

Iran is among moderate to high prevalence countries of multiple sclerosis (MS).^1^ According to a study on patients with MS in Tehran (capital of Iran), the annual percent change in registration of new cases during 1991 to 2014 was 12.8% and 12.5% in women and men, respectively.^2^ Since MS mostly affects young adults of 20-40 years old, Iranian public health system is going forward an increasing burden in near future.^3^ In a cost study of MS in Iran, the average cost per patients has been estimated around 296 United States dollars (USD).^4^

The major goal of MS treatments is to prevent or delay long-term disabilities,^5^ and interferon beta (INF-beta) is one of the most common treatments in the relapsing courses of disease. Patients’ compliance to interferon is however influenced by adverse effects such as flu-like symptoms, injection site reaction, and anxiety before injection.^6^^-^^8^

Recently, a pegylated (PEG) form of interferon beta 1a has been approved by the United States Food and Drug Administration (USFDA) as a new treatment for patients with MS. The most important advantage of this form is the less frequent administration which may leads to better patient compliance. Hence, due to the higher price of PEG-interferon beta 1a, it seems necessary to conduct an economic evaluation in order to make an evidence-based decision on it. There are several economic evaluation studies on interferon beta 1a in MS around the world,^9^^-^^11^ and also in Iran;^12^^,^^13^ but we did not find any cost-effectiveness analysis on PEG-interferon beta 1a at the time of this study. 

The aim of this study was to evaluate the cost-effectiveness of PEG-interferon beta 1-a compared with interferon beta 1a in Iran. This study was conducted as it was required by Iran FDA for registration of PEG-interferon beta 1 in Iran Drug List (IDL).^14^

## Materials and Methods

This study was an economic evaluation to compare total costs and effectiveness of both treatments in patients with MS. A hypothetical cohort of 1000 patients with relapsing remitting MS (RRMS) was entered to the model. The mean age of patients was considered as 37 years similar to clinical trials. This hypothetical cohort could receive either interferon beta 1a or PEG-interferon beta 1a once weekly versus twice monthly, respectively. For patients who had not responded to treatment or progressed to higher disability states, this treatment discontinued, and supportive care was prescribed. 

**Figure 1:**
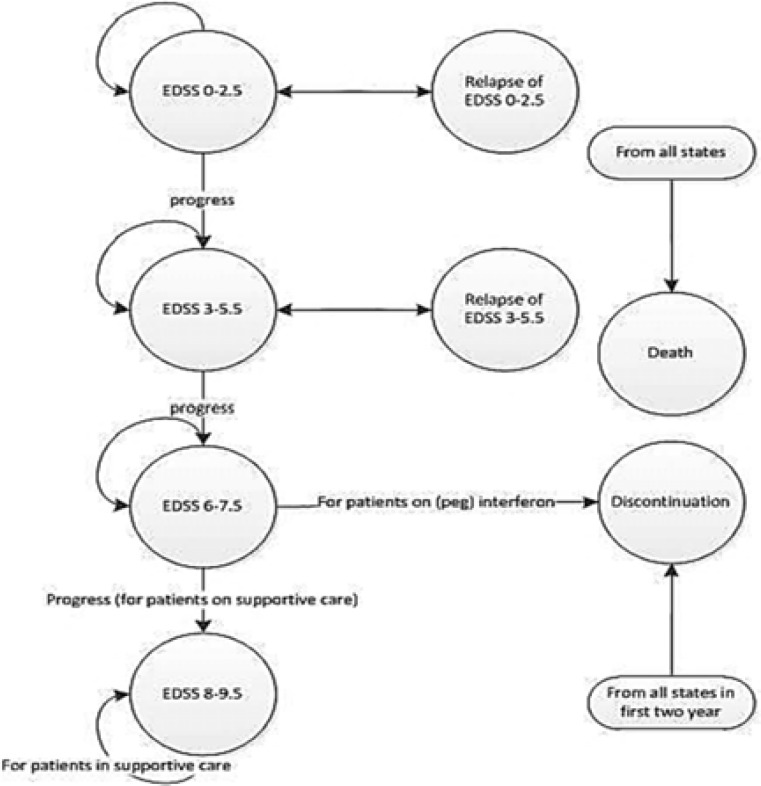
Schematic Markov model

***Model structure:*** Because of chronic nature of the disease, and frequent transition of patients between different levels of disability, a Markov model was selected to design a model to assess cost-effectiveness of using PEG-interferon beta 1a compared with currently used interferon beta 1a in patients with RRMS. The model was consisted of 8 states based on the Expanded Disability Status Score (EDSS), developed by Kurtzke,^15^ as a measure of quantifying disability in MS. Accordingly, four health states including EDSS of 0-2.5, 3-5.5, 6-7.5, and 8-9.5 were defined indicating low to severe disability levels, respectively. In addition, two temporary relapse states (relapse from EDSS 0-2.5, and relapse from EDSS 3-5.5), death, and treatment discontinuation were included. All patients started their treatment in EDSS 0-2.5, and those who discontinue treatment at any state would be switched to best supportive care (BSC) arm (Figure 1). Model cycle was 1-month length, and the time horizon was 10 years. This approach was followed in economic evaluation studies of MS by Lee, et al,^16^ Sanchez-de la Rosa, et al,^17^ and Prosser, et al.^18^

For model simplification, we assumed that relapse could only occur in two states, EDSS 0-2.5 and EDSS 3-5.5, and after that, no relapse would be possible. This assumption was consistent with current clinical observations and also with most of other economic evaluation studies.^16^^-^^21^ In addition, we assumed that no progression happened in relapse states, and tolerability-related discontinuation could only happen in the first four cycles.^22^^,^^23^

EDSS: Expanded Disability Status Score

***Transitional probabilities:*** To extract the transitional probabilities of patients between different states, the combination of published randomized clinical trials (RCTs), systematic reviews, meta-analysis (direct and indirect), and natural history data were used, given the lack of any head to head trial.^24^^-^^29^

**Table 1:** Transitional probabilities used in the model

**Base case**		**Base case**	**Reference**
Natural history of annualized progression rate			27-29
EDSS score	0-2.5	0.004438	
	3-5.5	0.009189	
	6-7.5	0.003583	
	8-9.5	0.000952	
Natural history of annualized relapse rate		0.078502228	27-29
Disease progression			
RR for PEG-interferon vs interferon beta 1a		0.629	24
RR for interferon beta 1a vs placebo		0.67	25
Relapse rate			
RR for PEG-interferon vs interferon beta 1a		0.851	24
RR for interferon beta 1a vs placebo		0.97	25
Withdrawal rate			
PEG-interferon beta 1a (annual)		0.046	33
Interferon beta 1a (2 years)		0.054	34

EDSS: Expanded Disability Status Score; RR: Relative risk, PEG: Pegylated

To estimate the age-dependent probability of mortality in patients with MS, Iran life table was used to extract the mortality rate of Iranian general population in different age groups; then, we calculated MS-adjusted age-dependent mortality probabilities using the relative risk of mortality of different EDSS states in population of patients with MS, reported by Danish MS registry.^30^^-^^32^ The probabilities used in this study are presented in table 1.

***Utility and quality-adjusted life-years (QALY):*** The utility score of each disability states and disability scores of relapses (0.42) estimated by Nikfar, et al for Iranian MS population were used for this study.^12^ We considered caregiver disutility associated to each state based on Gani, et al.^31^ More details about utility score are provided in table 2.

***Cost analysis and discounting:*** Direct medical and non-medical costs were included in the analysis from a payer perspective (patients and third party payers). The Iran’s pharmaceutical price list was used to calculate medicines cost. We discounted costs of following years with 7.2% as suggested by Abdoli in Iran.^35^ More details about costs are presented in table 3. 

All the calculations was done with currency rate of 29000 Iranian Rial (IRR) to 1 USD in 2016**.**

## Results

***Base case Analysis:*** According to our analysis, total discounted cost in PEG-interferon was 68,688 USD; while total discounted cost in interferon arm was estimated 59,308 USD. In each arm, cost of PEG-interferon and interferon beta 1a were around 99% and 97% of total cost, respectively. The total discounted QALY in PEG-interferon and interferon were 5709.88 and 4865.61, respectively. In other words, the estimated incremental cost-effectiveness ratio (ICER) in this study was 11,111 USD per QALY gained for the PEG-interferon compared with interferon regimen. Compared to 5,315 USD and 15945 USD as cost effectiveness threshold, PRG-interferon could be considered a cost-effective strategy in Iran. 

**Figure 2:**
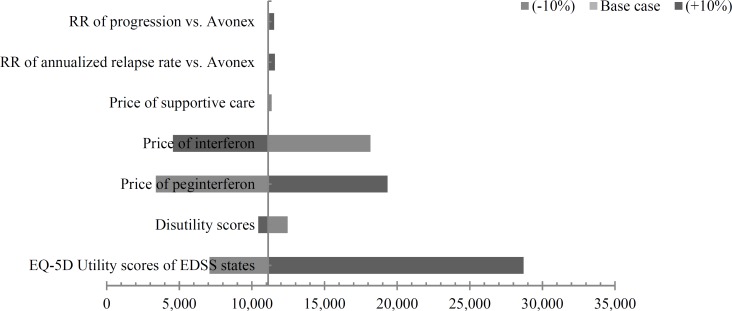
Tornado diagram for sensitivity analysis

***Sensitivity analysis:*** One-way deterministic sensitivity analysis results are reported in figure 2 and table 4 as tornado diagram and scenario analysis, respectively.

**Table 2:** Utility and disutility scores according to different health states

**Disability state**		**Patients utility and disutility (EQ-5D)**	**Caregiver disutility**
EDSS score	0-2.5	0.76	0
	3-5.5	0.56	-0.01
	6-7.5	0.21	-0.04
	8-9.5	-0.01	-0.12
Death		0	Not applicable
Relapse		-0.42	Not applicable
Adverse effect		0	Not applicable
Twice injection		-5% of each state’s utility	Not applicable

EQ-5D: EuroQol 5 Dimentions; EDSS: Expanded Disability Status Score

**Table 3:** Medical and non-medical direct costs

**Treatment-related ** **costs**	**PEG-** **interferon**	**Interferon**	**Supportive ** **care**	
Medicines	828 USD	695 USD	9 USD	
Injections	0	2.75 USD	1.72 USD	
State-related costs	EDSS of 0-2.5	EDSS of 3-5.5	EDSS of 6-7.5	EDSS 8-9.5
Physician visit	(1/6) × 4 USD	(1/6) × 4 USD	(1/3) × 4 USD	(1/3) × 4 USD
Psychotherapy	(1/6) × 4 USD	(1/6) × 4 USD	(1/6) × 4 USD	(1/6) × 4 USD
Rehabilitation	Not applicable	Not applicable	(2 times per week) × 4.5 USD	(2 times per week) × 4.5 USD
Nursing	Not applicable	Not applicable	172 USD	276 USD
Cost of relapse	172 USD	172 USD	172 USD	172 USD
One time per treatment costs*				
House reconstruction	3,448 USD			
Car rebuilding	1,724 USD			
Auxiliary instruments (cane, wheelchair, etc.)	69 USD			

USD: United States dollar; EDSS: Expanded Disability Status Score

* When a patient comes to EDSS state of 6-7.5

As it is depicted in figure 2, 10% increase in utility scores of all states had the most impact on ICER, while the relative risk of progression and relapse of PEG-interferon versus interferon had a negligible effect.

## Discussion

According to this study, at current price, PEG-interferon beta 1a could be considered cost-effective in Iran, compared with interferon beta 1a. Sensitivity analysis indicated that results were robust over most of key input variations. According to deterministic sensitivity analysis, PEG-interferon beta 1a was not a cost-effective strategy in Iran, in case of being compared with available lower price biosimilar form of interferon beta 1a with assumption of the equal efficacy. However, this assumption might be questionable.^36^

This study was the first cost-effectiveness analysis on PEG-interferon beta 1a compared with interferon beta 1a for treatment of MS in Iranian setting. We also could not find any published full economic evaluation on PEG-interferon in other countries. There was only one study published as poster presentation on the effect of using PEG-interferon on cost of hospitalization and relapses. According to this study, PEG-interferon beta 1a was expected to reduce hospitalization and relapse costs, respectively by 1297 and 1941 USD.^37^

We used a 10-year Markov model to consider long-term consequences of pegylated or non-pegylated forms of interferon beta 1a on a large hypothetical population of interest.

**Table 4:** Results of scenario analysis

**Scenario**	**Δ QALY**	**Δ cost (USD)**	**ICER (USD per QALY)**	**Interpretation**
Base case	844.27	9380845	11111	< 3 × GDP per capita
Cost discount rate (5%)	844.27	10199290	12080	< 3 × GDP per capita
Utility discount rate (3%)	918.99	9380845	10208	< 3 × GDP per capita
Interferon generic price	844.27	53249314	63071	> 3 × GDP per capita
Utility score system (VAS)	621.44	9592876	15436	> 3 × GDP per capita

USD: United States dollar; QALY: Quality-adjusted life years; ICER: Incremental cost-effectiveness ratio; GDP: Gross domestic product; VAS: Visual analogue scale

To make it a Markov model rather than a Markov chain, transition probabilities in each cycle were set to varying probability of death in each cycle, and linked to population mean age. However, mortality rate was only time-varying parameter in model, and other factors including previous cycle characteristics were not included here. In this study, transition probabilities were extracted from different available clinical evidences including RCTs, systematic reviews, direct and indirect meta-analysis, and natural history data. We also used Iranian utility score for each state from a recently published pharmacoeconomic study.

## Conclusion

According to this study, twice monthly PEG-interferon beta 1a could be considered as a cost-effective alternative for once weekly interferon beta 1a for Iranian patients with RRMS. 

However, by probable access to direct clinical evidences in future, more accurate clinical and economic judgment could be achieved.

## Notes:

**How to cite this article:** Hashemi-Meshkini A, Zekri HS, Karimi-Yazdi H, Zaboli P, Sahraian MA, Nikfar S. Pegylated versus non-pegylated interferon beta 1a in patients with relapsing-remitting multiple sclerosis: A cost-effectiveness analysis. Iran J Neurol 2018; 17(3): 123-8.
